# Rectal Carcinoma: Demographics and Clinicopathological Features from Pakistani Population Perspective

**DOI:** 10.7759/cureus.1375

**Published:** 2017-06-20

**Authors:** Muhammad T Pirzada, Monis J Ahmed, Anam Muzzafar, Irfan ul Islam Nasir, Muhammad F Shah, Shahid Khattak, Aamir A Syed

**Affiliations:** 1 Department of Surgical Oncology, Shaukat Khanum Memorial Cancer Hospital and Research Center, Lahore, Pakistan; 2 Department of Surgery, Mediclinic City Hospital, Dubai, UAE; 3 Surgery, Royal London Hospital

**Keywords:** clinicopathological features, demographics, rectal carcinoma, young age

## Abstract

**Background:**

Colorectal carcinoma is ranked as the second most common cancer diagnosis in females and third in males. It is the third leading cause of cancer-related deaths worldwide. Disease burden has been attributed to a myriad of factors comprising genetic, environmental, and dietary factors. Rectal cancer has been shown to demonstrate variance according to the geographical location.

**Methods:**

A retrospective review of 477 rectal cancer patients treated at Shaukat Khanum Memorial Cancer Hospital & Research Centre from 2006 to 2014 was performed. Demographic and clinicopathological features were compared between the two age groups (≤40 or >40 years). These included sex, ethnicity, family history of cancer, the location of tumor, clinical staging, histopathological type, and response to chemoradiation. Chi-square was used to compare the frequencies between the two age groups. p-value < 0.05 was taken as significant.

**Results:**

Mean age of the study group was 44.62 ± 16.11 years. 43.8% were ≤40 years of age, and 70.2% were male. 50.3% patients belong to Punjab province, 287 (60.2%) had lower rectal cancer, family history of cancer was present in 82 (17.2%) patients. 432 (90.5%) patients had T1/T2 disease and 296 (62.1%) had N2 disease. Metastatic disease at presentation was observed in 37 (7.8%). Progressive disease was found in 90 (18%) patients.

**Conclusion:**

High frequency of young onset rectal cancers and the lack of family history emphasize the need of indigenous strategies and national awareness of this disease for an early identification of these patients.

## Introduction

Colorectal carcinoma is ranked as the second most common cancer in females and third in males [1]. It is the third leading cause of cancer-related deaths worldwide [2]. Disease burden has been attributed to a myriad of factors comprising genetic, environmental, and dietary factors. Rectal cancer has been shown to demonstrate variance according to the geographical location [3].

Although cancer of rectum is considered to be a disease of elderly population, the occurrence is not uncommon in younger age group [4]. The risk of occurrence at a younger age is linked to the genetic arm of causation and has led to the development of many screening tools and criteria like Amsterdam and Bethesda to identify the population at risk [5-6]. Furthermore, rectal cancer diagnosis in patients younger than 40 years of age has been reported as to carry a poor prognosis and advanced stage at presentation [7]. With the intention of reducing these untoward outcomes, there is a continuous need to analyze the descriptive epidemiology of this tumor with respect to different geographical locations. In this regard, a wealth of international literatures has already been showing a strong association of rectal cancer with low age group for two decades. A study conducted in Los Angeles demonstrated that 3.95% of rectal cancer patients belonged to age group less than 40 years having different ethnic background [8]. On a similar note, Keswani, et al., investigating from New Orleans, observed that only 3.6% of their patients were under the age of 40 years [9]. Contrary to these western data, Nath, et al. showed relatively high frequency (35.5%) of rectal cancer in younger Indian population [10]. In summary, these studies re-demonstrate that the presentation and behavior of this cancer is affected by the geographical, racial, and other factors.

In Pakistan, it has long been an observation that the rectal carcinoma patients are generally younger. A few sporadic studies have emerged over the years suggesting this fact. In a small series, this percentage was reported to be 26.3% in rectal carcinoma patients below 30 years of age group [11]. Another study reported the ratio of under age 40 to over age 40 patients in colorectal cancer to be 0.3 [12]. If this indeed is verifiable, the implication on National Health Policy will be of great magnitude. Therefore, we decided to retrospectively analyze the managed cases of rectal carcinoma at Shaukat Khanum Memorial Cancer Hospital & Research Centre, Pakistan with regards to demographical and clinicopathological features.

## Materials and methods

A retrospective review of patients having rectal cancer managed (surgically or non-surgically) at Shaukat Khanum Memorial Cancer Hospital & Research Centre, Pakistan was accomplished from January 2006 to December 2014. Clinical information was retrieved and those fulfilling the inclusion criteria were included in the study. As per protocol, exemption from Institutional Review Board of Shaukat Khanum Memorial Cancer Hospital & Research Centre has been granted due to retrospective nature of the study.

Exclusion criteria included the following; patients diagnosed with Familial Adenomatous Polyposis (FAP), patients with synchronous, metachronous or recurrent rectal carcinoma, patients with squamous cell carcinoma of anal canal and rectal gastrointestinal stromal cell tumors (GIST) and patients in whom presence of rectal involvement secondary to prostate, bladder, uterus, cervix and vaginal tumor was present.

Patient’s demographic and clinicopathological features were compared between the two age groups (≤40 or >40 years). These included sex (male or female), ethnicity (Punjab, Khyber Pakhtoon Khwa [KPK], Afghanistan, Sindh, and Balochistan), family history of cancer, the location of tumor, clinical staging and histopathological type, and response to chemoradiation. Numerical variables were expressed as mean (SD) while categorical data was summarized as frequency (%). Chi-square was used to compare the frequency of all variables between the two age groups. p-value < 0.05 was taken as significant.

## Results

A total of 477 patients fulfilling the inclusion criteria were identified by retrospective analysis of the registered cases. The mean age of the study group was 44.62 ± 16.11. 209 patients (43.8%) were ≤40 years of age, and overall 335 (70.2%) were male patients (Table 1). Overall results show 287 (60.2%) had lower rectal cancer and family history of cancer in 82 (17.2%) patients. 432 (90.5%) had T1/T2 disease and 296 (62.1%) had N2 disease. Metastatic disease at presentation was observed in 37 (7.8%). 178 (37.3%) had moderately differentiated and 113 (23.7%) had poorly differentiated tumors. Progressive disease was found in 90 (18%) patients (Table 1).

**Table 1:** Overall characteristics.

Characteristics	n = 477	%
Age		
<40 years	209	43.8
>40 years	268	56.2
Mean age (years)	44.62 ± 16.11	
Sex		
Male	335	70.2
Female	142	29.8
Location		
Upper	46	9.6
Mid	144	30.2
Lower	287	60.2
Positive family history of cancer	82	17.2
T stage		
T1/T2	45	9.5
T3/T4	432	90.5
N stage		
N0	77	16.1
N1	104	21.8
N2	296	62.1
Metastatic	37	7.8
Histology		
Well differentiated	77	16.1
Moderately differentiated	178	37.3
Poorly differentiated	113	23.7
Signet ring cell	59	2.4
Mucinous	43	9
Melanoma	07	1.5
Response to chemoradiation		
Complete response	28	5.9
Partial response	151	31.7
Stable disease	110	23.1
Progressive disease	90	18.9

Overall, the majority of patients (240; 50.3%) belonged to Punjab province followed by 148 (31%) patients from KPK, 38 (8%) from Afghanistan, 32 (6.7%) from Sindh, and 19 (4%) from Balochistan province. Overall 157 (33%) patients had bleeding per rectum as an exclusive symptom to consult a physician. 197 (41.3%) patients experienced a change in bowel habit along with bleeding per rectum and 123 (25.7%) patients reported a change in bowel habits along with weight loss as a primary symptom to visit a physician for treatment. Family history was found in 27 (12.9%) patients less than 40 years of age in comparison to 55 (20.5%) patients more than 40 years (p = 0.037). Metastatic disease at presentation is fairly equal in both groups that is, 14 (6.7%) patients <40 years vs 23 (8.6%) patients in the other group (p = 0.49). Response assessment; 57 (31.7%) patients <40 years had partial response on chemoradiation while 56 (31.1%) patients resulted in progressive disease in comparison to 94 (47.2%) and 34 (17.1%) of patients with advance age group, respectively (p = 0.002). Young age patients had more aggressive disease in terms of advance stage disease at presentation and poorly differentiated tumors; 93.3% patients had T3/T4 disease and 31.1% patients with poorly differentiated tumors in contrast to patients with age more than 40 years (Table 2).

**Table 2:** Comparison of clinicopathological characteristics among age groups.

Characteristics	Age <40 [n = 209]	Age > 40 [n = 268]	p-value
	n (%)	n (%)	
Sex			
Male	149 (71.3)	186 (69.4)	0.654
Female	60 (28.7)	82 (30.6)	
Location			
Upper	23 (11)	23 (8.6)	0.626
Mid	64 (30.6)	80 (29.9)	
Lower	122 (58.4)	165 (61.6)	
Family history of cancer	27 (12.9)	55 (20.5)	0.037
T stage			
T1/T2	14 (6.7)	31 (11.6)	0.008
T3/T4	195 (93.3)	237 (88.4)	
N stage			
N0	19 (9.1)	58 (21.6)	0.000
N1	34 (16.3)	70 (26.1)	
N2	156 (74.6)	140 (52.2)	
Metastatic	14 (6.7)	23 (8.6)	0.493
Histology			
Well differentiated	23 (11)	54 (20.2)	0.001
Moderately differentiated	70 (33.4)	108 (40.3)	
Poorly differentiated	66 (31.6)	47 (17.5)	
Signet ring cell	30 (14.4)	29 (10.8)	
Mucinous	16 (7.7)	27 (10.1)	
Melanoma	04 (1.9)	03 (1.1)	
Response to chemoradiation			
Complete response	11 (6.1)	17 (8.5)	0.002
Partial response	57 (31.7)	94 (47.2)	
Stable disease	56 (31.1)	54 (27.1)	
Progressive disease	56 (31.1)	34 (17.1)	

## Discussion

The extensive research into the pathogenesis of rectal cancer has led to the implication of a number of factors ranging from different genes responsible for the identification of pathways of pathogenesis including chromosomal instability and hypermethylation of the promoter of the MLH1 gene [13].

Family history and cancer syndromes were traditionally seen as the major if not the only reason for the ‘rare’ occurrence of colorectal cancer in the younger population. Hence traditional screening programs developed were focused on the elderly population with the age of onset of screening being lowered for the subgroup of population that had a strong risk on the basis of family history.

The issue of the rectal cancer patients in relation to their age was addressed in a recent population-based study [14]. The authors concluded that the younger population was associated with a more advanced stage of disease at presentation and T4 tumors and node positivity was seen more in this subgroup. They also concluded that despite an advanced stage younger patients had a better prognosis vs elderly population on a stage to stage basis. However, overall young age of onset is associated with a poorer outcome as reported by Dozois, et al. [15].

The distribution of rectal or colorectal cancer is not spread uniformly throughout the world and shows geographical variations with the highest rates in USA, Australia and lowest in Africa and Central Asia [16-17]. The proportion of patients being affected in the young age also shows geographical distribution. A Canadian study, evaluating the prognosis of younger population, took the cutoff age as 45 and reported 3.36% of their study patients to belong to this group [14]. Another study from the USA reported 11.3% of patients belong to young age group [18]. In contrast, a study from Sudan reports 17% of their patients to be <40 years and 43.84% to be <50 years [19]. This high proportion is also reported to be 40% by another study from India [20]. As this ratio of young patients presenting with rectal cancer is very similar to our observation it gives more weightage to the theory of geographical factors influencing the appearance or presentation of rectal cancer.

The result of this study showed that 43.8% of the patients were under the age of 40 years. The obvious determination can be that of familial genes and syndromes being prevalent in these patients presenting with early onset cancer. However, family history (liberally defined as any cancer in the family members) was positive in only 17.2%. This puts a severe question to the applicability of using family history as a guide to risk assessment of having this cancer at a young age.

It is already known that rectal cancer specifically and cancers, in general, have a better outcome if caught early and intervened upon. Unfortunately, as seen in our study overall 90.5% of the patients had T3/T4 tumors, while 62% had N2 disease and 7.8% were metastatic at presentation. Moreover, lack of family history, partial response to chemoradiation and progressive disease on chemoradiation in younger age highlight a dire need of identification and implementation of national policies for the early identification and management of these patients. To the best of our knowledge, this is the single largest published from Pakistan.

## Conclusions

The frequency of rectal cancer is increasing in young age in Pakistani population. The high frequency of young onset of rectal cancer and the lack of family history emphasize the need of indigenous strategies and national awareness for an early identification of these patients.
